# Evaluation of CD38 expression in Sudanese patients with chronic lymphocytic leukemia

**DOI:** 10.1186/s13104-018-3926-0

**Published:** 2018-11-15

**Authors:** Enaam Abdelrhman Abdelgader, Nada Hassan Eltayeb, Tasniem Ahmed Eltahir, Osama Ali Altayeb, Eman Abbass Fadul, Eldirdiri M. Abdel Rahman, Tarig H. Merghani

**Affiliations:** 1 Department of Pathology, Faculty of Medicine, Al Neelain University, Khartoum, Sudan; 2 Department of Physiology, Faculty of Medicine, Al Neelain University, Khartoum, Sudan; 3 Khartoum Radioisotope Center, Khartoum, Sudan; 4 Flow Cytometry Laboratory for Leukemia & Lymphoma Diagnosis, Khartoum, Sudan; 5 Department of Physiology, Faculty of Medicine, University of Khartoum, Khartoum, Sudan

**Keywords:** CD38, Leukemia, CLL, Flowcytometer

## Abstract

**Objective:**

The objective of this study was to evaluate the cluster of differentiation-38 (CD38) expression in Sudanese patients with chronic lymphocytic leukemia (CLL) and to determine its association with clinical and laboratory characteristics of the disease.

**Results:**

We conducted a cross-sectional study on 99 patients diagnosed with CLL in Khartoum Oncology Hospital in Khartoum, Sudan. Immunophenotyping and CD38 expression levels were measured with four-color flowcytometry. The results of physical examination and blood analyses were used for assigning a modified Rai clinical staging system. The collected data were analyzed using the Statistical Package for the Social Science, version 22 (SPSS Inc., Chicago, IL, USA). According to our findings, the frequencies of 7%, 20%, and 30% cutoff levels of CD38 expressions were 68.7%, 41.4%, and 36.4% respectively. CD38 cutoff level of 7% showed a significant association with hemoglobin concentration (P = 0.04), whereas other cutoff levels showed insignificant results. All the three cutoff levels showed insignificant associations with the other clinical and laboratory variables. In conclusion, the CD38 expression at a cutoff level of 7% seems to be more valuable clinically than higher cutoff levels in Sudanese CLL patients.

## Introduction

Chronic lymphocytic leukemia (CLL) is a chronic lymphoproliferative malignancy characterized by an accumulation of cluster of differentiation-5 (CD5) ‏ monoclonal B cells in both primary and secondary lymphoid tissues [1]. The distinct immunophenotype of CLL lymphocytes is essential to confirm the diagnosis [2]. The 1994 scoring system was based on the evaluation of five parameters: CD5, CD23, FMC7, an intensity of kappa/lambda chains, and CD22/CD79b. The B-CLL score would range between five (typical B-CLL cases) and three (less typical B-CLL cases). Lower scores (0–2) would exclude the diagnosis of B-CLL [3].

CD38 is a type II transmembrane glycoprotein that acts as a complex ectoenzyme, a receptor molecule, and a signaling factor in lymphocytic cells [4]. It participates in many cellular activities that include cell adhesion, signal transduction, and calcium regulation [5]. CD38 expression, as well as zeta-chain-associated protein kinase-70 (ZAP70) and the mutation status of immunoglobulin variable region heavy chain (IgVH), are important prognostic indicators in CLL. The advantage of CD38 is its stability over time and its easy measurement [6, 7].

The expression of CD38 by CLL cells is associated with an aggressive clinical presentation that is confirmed by many studies [8–13]. The progression-free intervals in CD38 +ve patients are shorter, and they die sooner when compared with the CD38 −ve patients [8]. This observation encouraged many clinical centers to adopt the determination of CD38 percentage expression as part of the regular investigations of CLL patients [14].

Flowcytometry is a useful tool for population analysis. It enables measurements of multiple characteristics in single cells. Light scattering can detect differences in size and internal complexity, and the fluorescence emitted from labeled antibodies can locate cell surface and intracellular antigens [15].

The analysis of both CD38 and ZAP-70 would provide valuable information in the diagnostic work-up of B-CLL patients [16]. CD38 expression also correlates with the IgVH mutation status [17, 18]; however, the two parameters were found to be independent prognostic factors in B-CLL [19, 20]. Besides, CD38 is an independent predictor of progression-free survival (PFS) in Binet stage A patients [21].

Many articles described the dynamic expression of CD38 that indicates the proliferative activity of the leukemic cells at the time of analysis [22, 23]. The expression could be a parallel indicator of clonal evolution, which ultimately determines the patient’s prognosis [24]. On the other hand, the best threshold for CD38 positivity is unclear. Early reports suggested 30% as a cutoff level of interest, but lower figures (7%, 20%) might be more clinically relevant [25].

There is a lack of data regarding the prevalence of CD38 positivity or its clinical application in the management of CLL patients in Sudan. We carried out this study to estimate the level of CD38 expression in CLL patients, to find its association with baseline clinical and laboratory parameters at presentation, and to determine a cutoff level for a CD38 appearance that is more likely to be associated with the clinical and laboratory findings of CLL patients.

## Main text

### Methods

We conducted a cross-sectional study at Khartoum Oncology Hospital/Sudan, during the period from September 2016 to February 2017. The study included 99 newly diagnosed, untreated B-CLL patients (69.7% males, 30.3% females), diagnosed according to the International CLL Workshop Criteria [26] and staged according to the modified Rai system [27]. A structured questionnaire was used to collect clinical and demographic data. Physical examination was performed to determine the dimensional diameters of lymph nodes in the neck, axilla, supraclavicular, inguinal, and femoral regions. The size of the liver and spleen were assessed by chest radiography and abdominal ultrasound. Three ml of peripheral blood (PB) were withdrawn from each participant, collected in EDTA tubes, and preserved at room temperature (22–24 °C). All the samples were processed within 6–24 h of collection. Complete blood count (CBC) was obtained by automated hematology analyzer (Sysmex XE-2100™, Kobe, Japan). Four color flowcytometer (COULTER EPICS XL-MCLTM Flowcytometer—Miami, Florida—USA) with SYSTEM II software was used to determine the immunophenotyping and CD38 expression for the study population. The instrument set up was checked daily using QC check beads Flowcytometry (Beckman Coulter, Miami, USA).

The immunophenotyping (IPT) of lymphocytes (from lysed whole peripheral blood samples) was carried out to confirm the diagnosis of CLL using the following monoclonal antibodies (Beckman Coulter); CD45, CD5, CD19, CD20, CD22, CD23, kappa and lambda light chains, FMC7, and CD79b. A marker was considered positive at a cutoff level of 20%. The Matutes scoring system allocates one point each for the expression of weak SmIg, CD5, CD23, and absent or low expression of CD79b and FMC7 [3, 28].

The CD38 was analyzed in peripheral blood using anti CD38 monoclonal antibodies (Mo Ab), (from Beckman Coulter, Miami, USA). The cell surface staining method was applied according to the following protocol: Twenty microliters labeled Mo Ab were dispensed into appropriately labeled tubes. A sample of 100 µl was added containing no more than 1 × 10^4^ leukocytes/ml. Each tube was vortexed for 5 s. The tubes were incubated at room temperature in a dark place for 10 min. One milliliter of the RBCs lysis solution was added to each tube and then incubated at room temperature in a dark place for 10 min. Centrifugation at 1500*g* (3200 rpm) for 3 min was done, 3.5 ml PBS added, and centrifugation at 1500*g* (3200 rpm) for 3 min repeated. Samples were suspended in PBS, tubes vortexed and analyzed using flowcytometer. CD38 was measured by flowcytometry and plotted against B cell marker CD19 expression. The CD38 expression was considered positive at three cutoff levels; 7%, 20%, and 30%.

The collected data were analyzed using the software program of the Statistical Package for Social science for Windows (SPSS), version 22 (SPSS Inc., Chicago, IL, USA). Both quantitative variables (mean and standard deviation) and qualitative variables (number and percentage) were described. Chi square test was used for Comparison of categorical variables. Statistical significance was defined for a P-value less than 0.05.

The study was approved by the Ethical Research Committee of the Sudan Medical Specialization Board (SMSB, 8/2016). Official letters were sent to the Director of Khartoum Oncology Hospital. Informed written consent was obtained from each participant before sampling. The study followed the general principles of medical research and the stated guidelines of the Ethical Committee.

### Results

Ninety-nine CLL patients, including 69 (69.7%) males and 30 (30.3%) females, participated in this study. Their mean age (SD) was 62.0 (11.14) year, with a median of 63 (range 36–95) years. The clinical, hematological and flowcytometry findings are presented in Tables 1 and 2. The majority of the enrolled patients presented with lymphadenopathy (71.7%) and splenomegaly (52.5%). Complete blood count results showed anemia (72.7%), and thrombocytopenia (40.4%). The study results showed that nearly two-thirds of the participants (64.7%) were a high-risk group, 30.3% were intermediate risk group, and only 5.0% were low-risk group (Table 1). Using the scoring system, the majority of our patients had a score of 5.0 (53.5%), followed by a score of 4.0 (31.3%), (Table 1).

**Table 1:** Characteristics of CLL patients

Parameter	Frequency no (%)	Parameter	Frequency no (%)
*Age*		*Hemoglobin (g/dl)* ^a^	
≥ 60	65 (65.7%)	Severe anemia	12 (12.1%)
< 60	34 (34.3%)	Moderate anemia	32 (32.3%)
*Sex*		Mild anemia	28 (28.3%)
Male	69 (69.7%)	No anemia	27 (27.3%)
Female	30 (30.3%)	*Leukocyte count (*× *10*^*3*^*/µl)*	
*LN (no of sites involved)*		≤ 50	33 (33.3)
Absent	28 (28.3%)	> 50	66 (66.6)
1 site	4 (4.0%)	*Modified Rai staging system*	
2 sites	10 (10.1%)	High risk group	64 (64.7%)
3 sites	2 (2.0%)	Intermediate risk group	30 (30.3%)
> 3 sites	55 (55.6%)	Low risk group	5 (5.0%)
*Splenomegaly*		*Scoring system*	
Present	52 (52.5%)	Score 3	6 (6.1%)
Absent	47 (47.5%)	Score 3.5	5 (5.1%)
*Hepatomegaly*		Score 4	31 (31.3%)
Present	25 (25.3%)	Score 4.5	4 (4.0%)
Absent	74 (74.7%)	Score 5	53 (53.5%)
*Platelets count (*× 10^3^/µl*)*		*CD 38 positivity*	
< 150	40 (40.4%)	At 7% cutoff level	68 (68.7%)
150–450	57 (57.6%)	At 20% cutoff level	41 (41.4%)
> 450	2 (2.0)	At 30% cutoff level	36 (36.4%)

Distribution of the most important clinical and laboratory findings. Nearly two-thirds of the participants are within the high-risk group at presentation

^a^WHO classification of anemia

**Table 2:** Flowcytometry findings in CLL patients

Variables	Mean ± SD	Median (range)
CD45	85.05 ± 13.09	88.4 (0–98.20)
CD19	88.39 ± 8.94	90.60 (49.70–99.70)
CD20	81.62 ± 15.25	87.60 (12.90–98.40)
CD22	10.71 ± 16.67	3.94 (0–78.10)
CD23	64.40 ± 25.65	71.0 (15–96)
CD5	71.81 ± 30.99	88.4 (1.44–99.0)
CD79b	23.08 ± 30.71	19.30 (0–94.90)
FMC7	9.86 ± 15.65	3.70 (0.04–92.60)
Kappa	8.05 ± 15.42	2.05 (0–92.30)
Lamda	5.90 ± 15.58	0.59 (0–86.10)

Mean and median expression of immunophenotyping markers in the study population

The diagnosis of B-CLL was confirmed by flowcytometry. It revealed a typical immunophenotype; (kappa (κ)/lambda (λ) staining) weak, CD5+, CD19+, CD20 weak, CD23+, FMC7 (a CD20 epitope) and CD79b were absent or weakly expressed) (Table 2).

There was marked variability in the percentage expression of CD38 (range 0.09–96.1), with a median 13.00 and mean (SD) expression of 36.6 (37.92). The CD38 expression at cutoff levels of 7%, 20%, and 30%, were 68.7%, 41.4%, and 36.4% respectively (Table 3). CD38 cutoff level of 7% showed a significant association with the different classes of hemoglobin concentration (P = 0.04). The association with the other variables (age, sex, lymphadenopathy, splenomegaly, hepatomegaly, TWBC, platelets, and modified Rai staging systems was statistically insignificant (P > 0.05, Table 3).

**Table 3:** Association between the three CD38 cutoff levels and the study variables

Variables	7% cutoff level			20% cutoff level			30% cutoff level		
	CD38+	CD38−	P value	CD38+	CD38−	P value	CD38+	CD38−	P-value
*Age (%)*									
≥ 60	46.5	19.2	0.64	28.3	37.4	0.41	25.3	40.4	0.53
< 60	22.2	12.1		13.1	21.2		11.1	23.2	
*Sex (%)*									
Male	51.5	18.2	0.09	31.3	38.4	0.28	27.3	42.4	0.39
Female	17.2	13.1		10.1	20.2		9.1	21.2	
*Lymphadenopathy (%)*									
Not found	20.2	8.1	0.49	11.1	17.2	0.68	10.1	18.2	0.37
One site	2.0	2.0		1.0	3.0		0.0	4.0	
Two sites	9.1	1.0		5.1	5.1		5.1	5.1	
Three sites	1.0	1.0		0.0	2.0		0.0	2.0	
More than three sites	36.4	19.2		24.2	31.3		21.2	34.3	
*Splenomegaly (%)*									
Present	35.4	17.2	0.76	22.2	30.3	0.86	20.2	32.3	0.65
Absent	33.3	14.1		19.2	28.3		16.2	31.3	
*Hepatomegaly (%)*									
Present	18.2	7.1	0.68	13.1	12.1	0.21	11.1	14.1	0.36
Absent	50.5	24.2		28.3	46.5		25.3	49.5	
*Hemoglobin concentration (g/dl) (%)*									
Severe	11.1	1.0	0.04*	6.1	6.1	0.67	6.1	6.1	0.57
Moderate	18.2	14.1		11.1	21.2		11.1	21.2	
Mild	17.2	11.1		11.1	17.2		8.0	20.2	
No anemia	22.2	5.1		13.1	14.1		11.1	16.2	
*TWBCs count × 10* ^*9*^ *(%)*									
< 50	26.3	8.1)	0.10	13.1	21.2	0.59	12.1	22.2	0.83
≥ 50	42.4	23.2		28.3	37.4		24.2	41.4	
*Platelet count × 10* ^*9*^ *(%)*									
< 150	26.3	13.1	0.94	16.1	23.2	0.97	14.1	25.3	0.71
≥ 150	42.4	18.2		25.3	35.4		22.2	38.4	
*Modified Rai staging system (%)*									
High risk group	44.4	20.2	0.54	26.3	37.4	0.10	18.2	46.5	0.99
Intermediate risk group	22.2	8.0		13.1	18.2		17.2	13.1	
Low risk group	2.0	3.0		2.0	3.0		1.0	4.0	

The CD38 expression at cutoff levels of 7%, 20%, and 30%, were 68.7%, 41.4%, and 36.4% respectively. CD38 (7%) showed the highest positive expression, and a significant association (*) with hemoglobin level

### Discussion

The CD38 is a glycoprotein expressed on the surface of many white blood cells, including lymphocytes of CLL, and it has been linked to aggressiveness and poor prognosis of the disease. The determination of CD38 expression and IgVH mutation status are ordinary laboratory investigations in CLL patients [29]. In the clinical setting, CD38 is an independent predictor of progression-free survival (PFS) rate in Binet stage “A” patients [30]. However, the cutoff value for CD38-positivity that is most likely to predict a poor prognosis has been a source of extensive discussion.

In our study, CD38 expression at cutoff levels of 7%, 20%, and 30%, were 68.7%, 41.4%, and 36.4% respectively. A major finding is a significant association between CD38 positivity at a cutoff level of 7% and hemoglobin concentration. Since the modified Rai staging system classifies CLL patients with hemoglobin concentration < 11.0 g/dl as a high-risk group, CD38 expression at a cutoff value of 7% could be more useful clinically in detecting the high-risk group than the other higher cutoff levels. Similar findings were described by Kröber et al. [31] and Falay et al. [32]. They reported that the cutoff value of 7% is an important prognostic marker that can be used in the classification of CLL patients into different prognostic groups.

The present study showed an insignificant association between CD38 positivity and many of the clinical and laboratory parameters that included age, gender, lymphadenopathy, splenomegaly, hepatomegaly, WBC count, platelet count, and modified Rai staging system. This might be related to the late presentation of most of our patients; as about two-thirds of them are a high-risk group at the time of diagnosis, 30% were an intermediate-risk group, and only 5% are a low-risk group. In contrast to our findings, a significant association was found between advanced disease and surface expression of CD38 in > 30% of B-CLL cells [29]. Contradictory results were also reported by Damle et al. who showed that high levels of CD38 (> 30%) are associated with unmutated IgVH genes [8], whereas the mutated IgVH genes that indicate good prognosis, were found in patients with < 30% CD38-positive cells. Our finding that CD38 positivity in CLL at a cutoff level of 30% was 36% goes in line with the published literature that reported comparable results [32–36].

In conclusion, the CD38 expression at a cutoff value of 7% is significantly associated with low hemoglobin concentration, and therefore, more likely to predict poor outcomes than the higher CD38 cutoff levels in Sudanese CLL patients. Further studies are highly recommended for determining the “progression-free survival” and the most likely predictors of prognosis in Sudanese patients.

## Limitations

The present study has many limitations. The sampling method that depended on the voluntary participation of participants, the late presentation of our patients compared to other studies, and the lack of follow-up information, should all be considered for interpretation of the results. The follow-up information is particularly relevant because it gives valuable information about the prognosis regarding survival rate and response to treatment.

## Abbreviations

CLL: chronic lymphocytic leukemia
CD: cluster of differentiation
IgVH: immunoglobulin variable region heavy chain
ZAP70: zeta-chain-associated protein kinase-70
TWBCs: total white blood cells count
PFS: progression-free survival
